# Behavioral Health and Response for COVID-19

**DOI:** 10.1017/dmp.2020.180

**Published:** 2020-05-29

**Authors:** Tonya Cross Hansel, Leia Y. Saltzman, Patrick S. Bordnick

**Affiliations:** Tulane University School of Social Work, New Orleans, Louisiana

**Keywords:** behavioral health, community mental health services, COVID-19, pandemics

## Abstract

Research from financial stress, disasters, pandemics, and other extreme events, suggests that behavioral health will suffer, including anxiety, depression, and posttraumatic stress symptoms. Furthermore, these symptoms are likely to exacerbate alcohol or drug use, especially for those vulnerable to relapse. The nature of coronavirus disease 2019 (COVID-19) and vast reach of the virus, leave many unknows for the repercussions on behavioral health, yet existing research suggests that behavioral health concerns should take a primary role in response to the pandemic. We propose a 4-step services system designed for implementation with a variety of different groups and reserves limited clinical services for the most extreme reactions. While we can expect symptoms to remit overtime, many will also have longer-term or more severe concerns. Behavioral health interventions will likely need to change overtime and different types of interventions should be considered for different target groups, such as for those who recover from COVID-19, health-care professionals, and essential personnel; and the general public either due to loss of loved ones or significant life disruption. The important thing is to have a systematic plan to support behavioral health and to engage citizens in prevention and doing their part in recovery by staying home and protecting others.

The coronavirus disease 2019 (COVID-19) pandemic has presented in nearly every country, with the United States currently centering as the epicenter of the virus. From the first case reported to the World Health Organization on December 31, 2019, to the total cases rapidly approaching 1 million in April 2020,^1^ the spread, lack of treatment or vaccine, and medical shortages are creating strains on the health-care system. Not only is the health-care system strained, but human systems are also suffering. The Center for Disease Control and Prevention’s suggested method to prevent virus transmission is physical distancing,^2^ resulting in a multitude of economic and social consequences. The unprecedented toll COVID-19 will take on individuals behavioral health is largely unknown. The purpose of this study is to provide a framework for the possible behavioral health outcomes based on research following other financial crises, disasters, and pandemics.

## FINANCIAL CRISIS

Recession depression, as noted by McInerney et al.,^3^ is the subjective mental health consequences following loss of wealth. Increases in depression are also evident in numerous studies noting associations among suicide rates and layoffs, unemployment, and financial crises.^4–6^ Conversely, Yin and colleagues^7^ found that improved gross domestic product resulted in decreased suicide rates. McLaughlin and colleagues^8^ found that home foreclosure due to reduced income resulted in increased rates of depression. Following the Greece debt crisis in 2009, Drydakis^9^ conducted a longitudinal study and found poorer mental health among those experiencing unemployment. Stein and colleagues^10^ found that economic constraints resulted in increased symptoms of depression and anxiety. Perhaps the most salient research is a study by McDaid and colleagues,^11^ suggesting that recessions can lead to increases in communicable diseases and suicide rates. In looking at the mental health effects of COVID-19, a highly infectious disease, and the early financial crises, mental health problems are likely to affect a larger portion of the population than the disease itself.

## DISASTERS

In the past 2 decades, disaster mental health research has increased greatly due to the subsequent increases in large scale disasters. Norris and colleagues^12,13^ published a compilation of disaster research through 2001, where they found common outcomes postdisaster include depression, anxiety, and posttraumatic stress. These symptoms were found most notably in communities of resources loss and were most prevalent in the year following disaster; however, it was noted that for some groups and disasters, symptoms may persist for years. Following Hurricane Katrina, an unprecedented natural disaster of its time in 2005, where survivors reported higher estimates of serious mental illness compared with previous surveys.^14^ Importantly, suicide rates remained low and authors suggest the protective factors of perseverance might have buffered the traumatic experience.^14^

Perhaps the most notable recent disaster where physical, mental, and financial problems combined was in the 2010 Deepwater Horizon oil spill in the Gulf of Mexico. Technological disasters have unique impacts, such that effects are often unknown, contributing to a lack of closure or return to predisaster state.^15^ For oil spills, the indirect or secondary exposure from job loss, economic impacts, litigation, and ongoing disruptions in daily life^16–18^ contributes significantly to the health of community members due to social and economic uncertainties that have significant consequences for behavioral health.^19,20^ Research following oil spills, including the Deepwater Horizon oil spill, have found increased rates of anxiety, depression, and posttraumatic stress symptoms.^19,21–23^ Osofsky and colleagues^22^ also found that existing traumas from Hurricane Katrina exacerbate oil spill-related symptoms and that increased symptoms maintained for 2 y post-spill.^24^ While COVID-19 is presenting as a type of bio-disaster, mental health outcomes are likely to be similar to other types of disasters.

## PANDEMICS

For over a decade, scholars have been calling for systematic plans for pandemic mental health response.^25^ Like disasters and financial crises, pandemics create a large amount of disruption, uncertainty, and public fear, which given other factors can lead to mental health problems. Following the severe acute respiratory syndrome (SARS) epidemic in 2003, females had more emotional disturbance; more education, belief in government efficacy, and accurate transmission knowledge resulted in less disturbance.^26^ Lau and colleagues^26^ also found that increased work, family, and financial stress were associated with worsened mental health. Mak and colleagues^27^ suggested that the SARS epidemic had both immediate and long-term consequences for mental health. They found persistent and elevated posttraumatic stress symptoms for the mental health of survivors and health-care workers almost 3 y post-event.^27^

Not only is mental health important for individual well-being, but it also plays a role in collective prevention and risk. Betancourt and colleagues^28^ found that, during the Ebola outbreak, depression and posttraumatic stress symptoms were associated with higher risk behaviors. Of interest, higher anxiety resulted in more preventative behaviors,^28^ demonstrating a potential factor of mild anxiety. Supporting these research findings, Reardon^29^ interviewed a mental health professional working in Sierra Leone. The responder noted high levels of anxiety and that “The majority of psychological problems are because the country is frozen, with nothing moving forward.” Not only should we expect there to be long-term sequela for survivors and health-care workers, but also for the general public, especially those that lost someone due to COVID-19.

## TRAUMATIC AND UNRESOLVED GRIEF EVENTS

Massive loss of life is a significant factor that impacts the mental health of individuals, families, and communities in disasters. Pandemics by their nature, result is high mortality rates that cluster in geographical regions over a short period of time. In the context of COVID-19, the mortality rate based on the current number of deaths and infections is approximately 3%,^1^ with modeling from the University of Washington projecting 82,000 deaths in the United States by August of 2020.^30^ Recent evidence suggests that the death rate is disproportionality high among racial and ethnic minority groups, possibly because of health disparities (pre–COVID-19) that leave these groups medically vulnerable and less able to participate in social distancing practices.^31,32^ The mental health implications resulting from widespread loss of life will be extensive and long lasting.

The public safety measures associated with COVID-19 containment create additional complexity surrounding the process of death, dying, and mourning. More specifically, family members may experience anticipatory grief and components of ambiguous loss; both are factors known to be associated with worse outcomes. Anticipatory grief refers to mourning the potential loss of a loved one before they die.^33^ Similarly, ambiguous loss refers to the uncertainty about the circumstances surrounding the death of a loved one.^34^ While research on grief and mental health conditions in previous pandemics is sparse, 1 study conducted in China following the H1N1 pandemic, demonstrated a negative association between being sick or having a family member who was sick with the development of posttraumatic stress symptomology.^35^ However, this study’s sample only consisted of 22 respondents who met criteria for posttraumatic stress disorder (PTSD) and only 2 of those who had been infected with H1N1. Furthermore, the study did not assess respondents for anticipatory grief reactions. The methodological flaws of this study leaving the generalizability of these results to individuals directly impacted by a pandemic virus, or those struggling with anticipatory grief in question. Other research exploring grief reactions in mass causality events have primarily focused on human-made disasters such as 9/11, again limiting their applicability to the context of COVID-19.^36^

Despite these gaps in the empirical literature, conceptually trauma and loss of life in mass casualty events are inextricably linked. For example, the criteria for PTSD outlined in the Diagnostic and Statistical Manual of Mental Disorders, 5^th^ Edition (DSM-5) specifically mentions exposure to an event in which death or threatened death of one’s self or a loved one as a form of trauma exposure.^37^ This link is perhaps even more significant in the context of COVID-19 because of the high mortality rates. As such, it is appropriate to expect an increase in the number of individuals who may meet criteria for PTSD following COVID-19. However, individuals who become sick and face the possibility of dying, and those who lose a loved one to COVID-19 are not the only populations at risk for PTSD.

Although difficult to parcel out given the highly contagious nature of COVID-19, many individuals (regardless of loss) will not become infected with the virus. There is a significant gap in the literature regarding the rates of PTSD in noninfected individuals during global pandemics. Research has explored this issue as it relates to survivors of the infection and front-line responders. One study, conducted by Sprange and Silman,^38^ did explore PTSD in noninfected populations, and found higher than average rates of PTSD in both children and parents in isolation or quarantine during a pandemic (25% and 30%, respectively). This dearth in literature suggests a need for further population-based research with noninfected subjects.

The DSM-5 criteria not only include the actual or threatened death of a loved one, but also includes the potential of losing one’s own life. This is particularly important in the context of COVID-19 as the rates of severe illness and hospitalization is approximately 4.6 per 100,000 of all COVID-19 patients^39^; while the mortality rate is currently approximately 3%.^1^ As such, most of the individuals who become critically ill will survive COVID-19. The general literature regarding the relationship between intensive care unit (ICU) hospitalization and PTSD is well established.^40^ The research suggests that PTSD among ICU survivors is fairly common and long lasting.^41,42^ Previous studies have demonstrated a link between hospitalization during a pandemic with PTSD.^43^ For example, in a study of long-term outcomes following the H1N1 pandemic in 2009, researchers found that at 1-y follow-up, between 41 and 44% of discharged ICU patients were at risk for PTSD.^44^ Similarly, a long-term follow-up of SARS survivors found that PTSD persisted in some patients even 30 months following the illness.^45^

Not unlike ICU patients, medical personnel and front-line responders have a unique profile of risk in the context of COVID-19. Front-line workers are exposed to the virus and potential illness at higher rates, did not have proper equipment and protocols in place to address pandemics, and are also likely to witness massive loss of life in the context of their jobs. While the burden of COVID-19 on the medical system is unparallel, previous research on front-line workers during SARS and Ebola does suggest that front-line workers face additional risk of negative mental health outcomes.^46,47^ Front-line workers may also experience compassion fatigue, the reduced ability to be empathic toward the suffering of patients during disasters or times of crisis.^48,49^ Previous research on the mental health consequences of the Ebola pandemic suggests that healthy coping strategies play a central role in mitigating the impact of negative mental health sequela; conversely, negative coping strategies, such as substance use, can exacerbate or lead to worsened outcomes.^50^

## EXTREME EVENTS AND SUBSTANCE USE

In general, substance misuse and abuse occur at times of increased stress and anxiety. Negative mood states (stress, anger, anxiety, depression) have been associated as risk factors for relapse to alcohol^51–53^ and other drugs of abuse. As such, attention needs to focus on the impact of long-term crises and disasters, which place individuals and communities at increased risk of substance misuse and relapse.^54–57^ Over the past 30-plus years, research has focused on substance use and misuse pre- and post-disasters. Numerous studies have had mixed results from reported increases in substance abuse and relapse postdisaster,^58–62^ to negative results.^63^

Importantly, behavioral health effects learned from the 2010 Deepwater Horizon oil spill resulting from loss of jobs, lack of confidence in systems, and loss of one’s current and future way of life,^64^ need to be addressed as these will be realized world-wide during and post–COVID-19 pandemic. In 2013, a report comparing data from the National Survey on Drug Use and Health (NSDUH) from 2007 through 2011 indicate that alcohol and marijuana use increased from pre-spill to post-spill levels across various age groups. However, cigarettes and illicit drug use remained at similar levels pre and post.^64^ Gould and colleagues,^65^ summarized these findings and postulated that key indicators, including perceived or real loss of income, loss of confidence in authorities, and loss of one’s culture or way of life, were related to significant behavioral health impacts, including substance misuse.

Overall, there is less literature on substance use and extreme events, but the connection between mental health and substance use problems is well evident. The 1 point that appears to be a consensus among the literature is individuals with current substance use diagnoses or currently in recovery are at an increased risk for relapse.^66,67^ Regardless of whether exposure to a disaster represents the emergence of a new substance misuse diagnosis or an exacerbation of pre-existing use,^67^ there is an emergent need to understand the magnitude and impact of these negative factors on substance misuse and relapse from COVID-19. Furthermore, it is important to consider both relapse and increased use in behavioral health services offered during and post-pandemic.

## COVID-19 BEHAVIORAL HEALTH RESPONSE AND PUBLIC HEALTH IMPLICATIONS

The COVID-19 pandemic has exposed hundreds of millions of individuals to the following: (1) job losses overnight and severe financial uncertainty; (2) instilled a lack of confidence and trust due to daily mixed messages from authorities on health, safety, and security; and (3) increased fears and anxiety on what life will be like post-pandemic. When we compile research from financial stress, disasters, pandemics, and other extreme events, it is very clear that behavioral health will suffer, specifically anxiety, depression, and posttraumatic stress symptoms will be on the rise. Norris and colleagues^12^ recommend early interventions following extreme events, especially when coupled with large scale financial stress and threats to or loss of life. Behavioral health interventions will likely need to change overtime and that for some groups, these acute symptoms will be prolonged and require more intensive clinical interventions.^13^ Different types of interventions should also be considered for different target groups, such as for those who recover from COVID-19, health-care professionals and essential personnel, and the general public, either due to loss of loved ones or significant life disruption.

We are proposing a 4-step socially informed public health approach that is consistent with the multistep processes recommended by Screening, Brief Intervention, and Referral to Treatment (SBIRT).^68^ It is designed for implementation with a variety of different groups and reserves limited clinical services for the most extreme reactions. Given the scope of the COVID-19 pandemic, we are also proposing to add outreach efforts at the beginning for collaborations, communications, and psychoeducation. These types of systematic responses have been used extensively following extreme events and are flexible toward application in a variety of settings with differing resources.^69–74^

The first step focuses on *outreach*, beginning in the immediate response and through long-term recovery. Collaboration built on existing, prior, or localized and coordinated response efforts.^73^ This will help identify needs and ensure that services are not duplicated; collaborations will also help to ensure that response efforts are culturally and geographically informed. Early in the response phase, outreach, specifically communications and psychoeducation efforts, is very important. To address the increased stressors, there is a need to highlight the importance of debriefing sessions into shift protocols for medical personnel and first responders versus being an add-on or optional activity. The Centers for Disease Control and Prevention (CDC) recommend that communication messaging, “…to a severe pandemic should acknowledge different emotions that may arise among the community in addition to stressing the importance of helping others.”^75^

Proper messaging should be frequent, consistent, and factual; it should can also extend into outreach efforts and provide psychoeducation. Information that can alter “self-talk and help tasks can be important during the recovery phase of a severe pandemic.”^75^ Psychoeducation can present positive coping strategies, normalize mental health response and fluctuation over the course of recovery, acknowledge problematic coping such as alcohol problems, and reinforce the importance of self-care. For COVID-19 messaging on financial coping and recognizing in oneself and loved one’s prolonged grief and trauma symptoms should also be provided. In addition, reinforcing the importance of community support, while maintaining social distancing is also important.

Once outreach has begun, the process of SBRIT begins with *screenings* to better understand the scope and type of behavioral health needs. Following disasters SBRIT focuses on community-wide screenings of both mental health and substance use problems. For community wide screenings, thresholds should be set low to identify not only problematic symptoms but risky behavior and most importantly to facilitate referral to services when warranted.^76^ In connection with screenings, *brief interventions* can be a follow-up to mild symptoms or for individuals that self-select for services above outreach. Brief interventions refer to, “time-limited effort (eg, 1-2 conversations or meetings) to provide information or advice and increase motivation to avoid substance use, or to teach behavior change skills,” (p. 8) beneficial to one’s mental well-being.^77^ Two evidence based brief service models useful post-disaster are Psychological First Aid^78^ and Skills for Psychological Recovery.^79^

The primary goal of step 3 is *referral to more intensive treatment*. Mental health professionals should be critical component of multidisciplinary efforts to manage public health post-pandemic.^27^ However, services are often in short supply after extreme events and should be reserved for when more than brief services are warranted.^80^ Factors associated with elevated symptoms, such as being female, preexisting conditions or traumas, substance use, suicide ideation, and personal safety considerations should also be considered for more intensive services. Persons also included in the need for more intensive services should be health-care workers, as these individuals have been noted in the literature for having longer-term posttraumatic stress symptoms.^81^

## SUMMARY

The nature of COVID-19 and vast reach of the virus, leave many unknowns for the repercussions on behavioral health, yet existing research suggests that behavioral health concerns should take a primary role in response to the pandemic. While we can expect many individuals with elevated symptoms or substance use problems to remit overtime, many will also have longer-term or more severe concerns, especially those with fewer resources and more life stressors.^82^ It is also important to consider how the greater population recovers; is it fully back to pre-existing states or are there continued mild symptoms that interfere with daily functioning. Unique to COVID-19 is the wide access to technology that may help buffer mental health problems, especially loneliness.^83^ Contrary to concerns, we may also see improved social cohesion, similar to studies following SARS.^26^ The important thing is to have a systematic plan to support behavioral health and to engage citizens in the prevention and doing their part in recovery by staying home and protecting others.
